# Effects of ulinastatin on cerebral oxygen metabolism and CRP levels in patients with severe traumatic brain injury

**DOI:** 10.3892/etm.2014.1666

**Published:** 2014-04-04

**Authors:** LEI HUI, FAZHENG SHEN, HAIGANG CHANG, XIANGSHENG LI, GUOJUN GAO, JIWEI MA

**Affiliations:** Department of Neurosurgery, The First Affiliated Hospital of Xinxiang Medical University, Weihui, Henan 453100, P.R. China

**Keywords:** ulinastatin, brain injury, cerebral oxygen metabolism, C-reactive protein

## Abstract

The aim of the present study was to investigate the effects of ulinastatin on cerebral oxygen metabolism and C-reactive protein (CRP) levels in patients with severe traumatic brain injury (sTBI). A total of 92 patients with sTBI, admitted to the First Affiliated Hospital of Xinxiang Medical University (Xinxiang, China), were randomly divided into control and observation groups. The control group received conventional therapy plus a placebo (0.9% sodium chloride), while the observation group were administered conventional therapy plus 200,000 units ulinastatin via intravenous injection twice a day for seven days. Arterial and jugular venous blood was collected for blood gas analysis. The jugular venous blood lactate (JVBL), jugular venous bulb oxygen saturation (SjvO_2_), arteriovenous oxygen content difference (AVDO_2_) and cerebral extraction of oxygen (CEO_2_) levels were measured on day 1, 3, 5 and 7, as well as the level of CRP in the peripheral blood. In the control group, the level of JVBL decreased as compared with the level at day 1, however, no statistically significant differences were observed (P>0.05). By contrast, the observation group exhibited a significant reduction in the level of JVBL (P<0.05), which was also significantly lower compared with the control group (P<0.05). Statistically significant differences were observed between the two groups with regard to SjvO_2_, AVDO_2_ and CEO_2_ on day 3, 5 and 7. The CRP levels in the two groups increased and peaked on day 3. However, the CRP level in the observation group significantly decreased on day 5 (35.27±15.18 mg/l) and day 7 (22.65±10.48 mg/l), which was lower compared with the control group (56.19±13.24 mg/l and 47.36±15.73 mg/l, respectively); statistically significant differences were observed (P<0.05). Therefore, ulinastatin effectively improved cerebral oxygen metabolism and reduced the CRP level in patients with sTBI.

## Introduction

The balance between cerebral oxygen supply and demand is necessary for the brain to maintain normal physiological activity. Patients with severe traumatic brain injury (sTBI) exhibit serious cerebral oxygen metabolism dysfunction at an early stage, which is a leading cause of mortality. Appropriate treatment for maintaining the cerebral oxygen supply-demand balance and reducing the relevant complications has great clinical significance (1). The body is in a state of inflammation following sTBI and the serum C-reactive protein (CRP) level increases rapidly, exacerbating the pathological damage (2,3). CRP has been reported to be a sensitive indicator that reflects cerebral trauma severity (4). Ulinastatin, a protease inhibitor, is predominantly used to treat acute and chronic pancreatitis. It has been reported that ulinastatin exhibits cerebral protective effects and may inhibit the release of inflammatory mediators, however, there is limited research on the use of ulinastatin in the treatment for sTBI (5). Therefore, the present study focused on the application of ulinastatin in the treatment of sTBI and investigated the effects of ulinastatin on cerebral oxygen metabolism and the level of CRP.

## Subjects and methods

### Subjects

In total, 92 patients admitted to the Intensive Care Unit (ICU) in the First Affiliated Hospital of Xinxiang Medical University (Xinxiang, China) between February 2010 and May 2013 with a clinical diagnosis of sTBI were enrolled in the study. Patients were included in the study if the following criteria were met: Diagnosis of sTBI by computed tomography or magnetic resonance imaging; Glasgow Coma Scale (GCS) score of <8; and admittance to ICU within 8 h after injury (6). Exclusion criteria included combined brain complications, combined severe injury and patient mortality within seven days following injury (7). A total of 92 patients with sTBI were randomly divided into two groups (n=46 per group). The male-to-female ratio, age range, average age and GCS score in the control group were 41:5, 28–63 years, 45.27±15.33 years and 5.49±0.71, respectively, while in the observation group, the parameters were 39:7, 25–61 years, 43.19±15.60 years and 5.52±0.74, respectively. The gender ratio, age and GCS score of the two groups exhibited no statistically significant differences (P>0.05). The study was conducted in accordance with the Declaration of Helsinki and with approval from the Ethics Committee of the First Affiliated Hospital of Xinxiang Medical University. Written informed consent was obtained from all the participants.

### Treatment

All the patients admitted to the ICU were administered conventional therapy, including ventilator-assisted therapy, oxygen therapy, intracranial pressure reduction, dehydration, bleeding control, neuroprotection and appropriate symptomatic treatments. The control group received conventional therapy plus a placebo (0.9% sodium chloride), while the observation group received conventional therapy plus ulinastatin (200,000 units; Techpool Bio-Pharma Co., Ltd, Guangzhou, China) via intravenous injection twice a day for seven days.

### Cerebral oxygen metabolism monitoring

Cerebral oxygen metabolism was monitored as previously described (8). Arterial and jugular venous blood was collected in the morning for blood gas analysis (ABL80; Radiometer Medical ApS, Copenhagen, Denmark). The parameters recorded included hemoglobin concentration (Hb), jugular venous blood lactate (JVBL), arterial oxygen saturation (SaO_2_), jugular venous bulb oxygen saturation (SjvO_2_), partial pressure of O_2_ in the arterial blood (PaO_2_) and partial pressure of O_2_ in the jugular venous blood (PjvO_2_). Parameters were calculated as follows: Arterial oxygen content (CaO_2_) = (Hb × 1.36 × SaO_2_) + (0.003 × PaO_2_); venous oxygen content (CjvO_2_) = (Hb × 1.36 × SjvO_2_) + (0.003 × PjvO_2_); arteriovenous oxygen content difference (AVDO_2_) = CaO_2_ - CjvO_2_; and cerebral extraction of oxygen (CEO_2_) = 1 - (CjvO_2_/CaO_2_).

### Assay of CRP level

Peripheral blood was collected from the patients on day 1, 3, 5 and 7 in the morning and the CRP levels were determined using an ELISA kit, according to the manufacturer’s instructions (Shanghai Hengyuan Biotechnology Co., Ltd, Shanghai, China).

### Statistical analysis

Data were analyzed using SPSS 15.0 software (SPSS, Inc., Chicago, IL, USA) and all the data are expressed as the mean ± standard deviation. Data were analyzed using the unpaired Student’s t test for comparisons between two groups and the χ^2^ test. P<0.05 was considered to indicate a statistically significant difference.

## Results

### Comparison of cerebral oxygen metabolism parameters between the two groups

**Figure 1:**
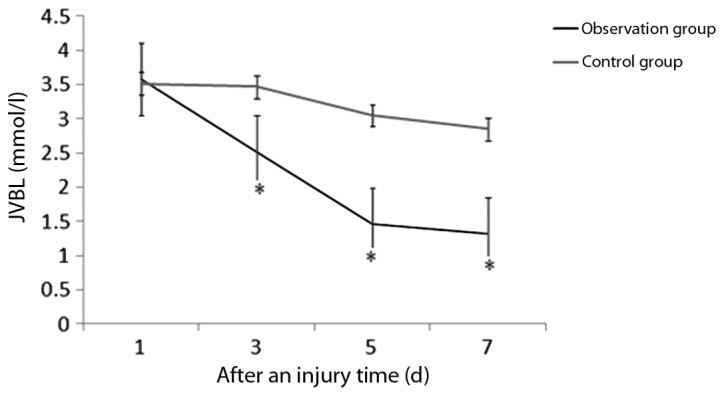
Comparison of JVBL concentration between the two groups. ^*^P<0.05, vs. control group. JVBL, jugular venous blood lactate.

**Figure 2:**
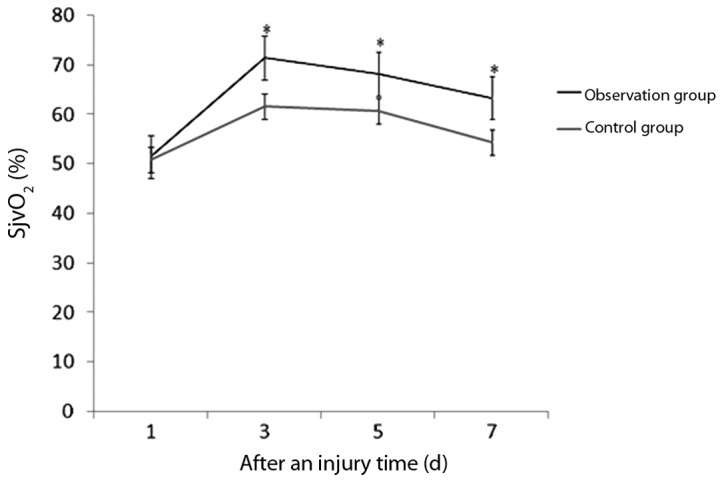
Comparison of SjvO_2_ between the two groups. ^*^P<0.05, vs. control group. SjvO_2_, jugular venous bulb oxygen saturation.

**Figure 3:**
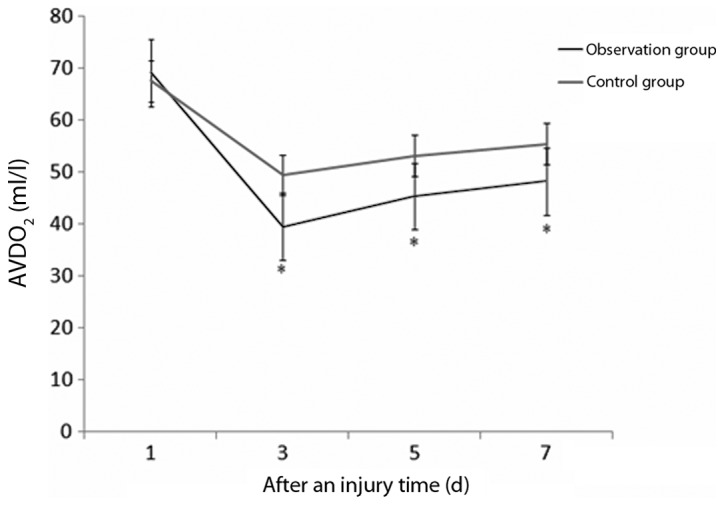
Comparison of AVDO_2_ between the two groups. ^*^P<0.05, vs. control group. AVDO_2_, arteriovenous oxygen content difference.

**Figure 4:**
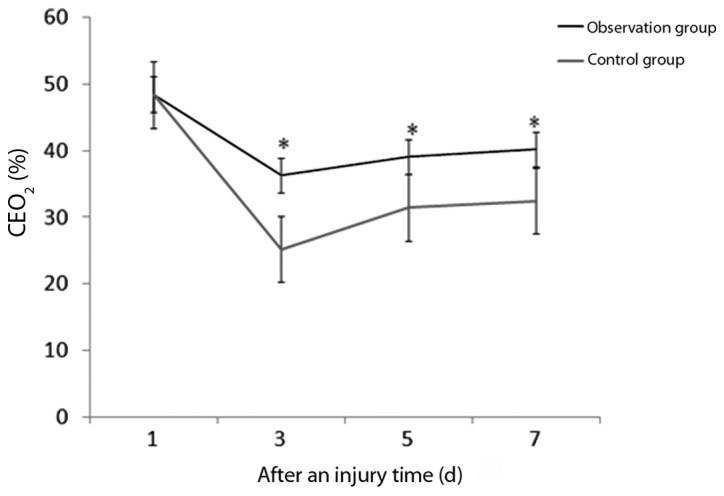
Comparison of CEO_2_ between the two groups. P<0.05, vs. control group. CEO_2_, cerebral extraction of oxygen.

JVBL, SjvO_2_, AVDO_2_ and CEO_2_ measurements for the two groups are shown in Table I. As shown in Fig. 1, the level of JVBL in the control group decreased as compared with the level at day 1, however, no statistically significant differences were observed (P>0.05). By contrast, the observation group exhibited a significant decrease in JVBL levels (P<0.05) when compared with the level on day 1, and the levels were also lower compared with the control group (P<0.05). The value of SjvO_2_ in the patients increased following injury and peaked on day 3. Statistically significant differences were observed between the two groups with regard to SjvO_2_ on day 3, 5 and 7 (Fig. 2). The AVDO_2_ level in the two groups decreased, and statistically significant differences were observed between the groups (Fig. 3). In addition, the CEO_2_ level in the two groups decreased, with the level in the control group decreasing significantly as compared with observation group (Fig. 4).

### Comparison of the CRP levels between the two groups

As shown in Table II, the CRP levels of the two groups increased and peaked on day 3 in the observation group. The CRP levels in the observation group significantly decreased on day 5 (35.27±15.18 mg/l) and day 7 (22.65±10.48 mg/l), and were lower than the values observed in the control group (56.19±13.24 mg/l and 47.36±15.73 mg/l, respectively); statistically significant differences were observed between the two groups (P<0.05).

### Comparison of gastrointestinal bleeding and mortality rates between the two groups

Gastrointestinal bleeding and mortality rates were also recorded for 30 days. In the control group, 20 patients (43.48%) showed gastrointestinal bleeding and 15 patients (32.61%) succumbed to their illness. In the observation group, only 11 patients (23.91%) showed gastrointestinal bleeding and eight mortalities (17.39%) were recorded. Statistically significant differences were observed when compared with the control group (P<0.05).

## Discussion

Cerebral ischemia-hypoxia occurs following the development of sTBI. Since the cerebral oxygen metabolism indicators, including JVBL, SjvO_2_, AVDO_2_ and CEO_2_, reflect the state of cerebral microcirculation and oxygen supply-demand, monitoring the associated indicators is an effective method for the early detection of cerebral ischemia and hypoxia (9). In addition, the body is in a state of inflammation with increased serum CRP levels following sTBI, and the serum CRP levels decrease when the body is recovered (10). Ulinastatin, as a protease inhibitor, has significant anti-inflammatory effects and has been used to treat pancreatitis. Recently, clinical studies have revealed that ulinastatin improves the circulation of shock caused by cell damage and exhibits cerebral protective effects (11). To date, there has been limited clinical use of ulinastatin in the treatment of sTBI. Thus, the present study aimed to investigate the effects of ulinastatin on cerebral oxygen metabolism and CRP levels.

A decrease of blood flow in cerebral circulation induces the brain tissue to draw oxygen from the blood at a higher proportion in order to maintain normal metabolism. By contrast, when the blood flow increases, the proportion of oxygen drawn from blood decreases, which results in increased oxygen content in cerebral circulation (12). Since the internal jugular vein is the main channel of cerebral circulation, blood gas analysis of the internal jugular vein indirectly reflects cerebral oxygen metabolism (elevated SjvO_2_ indicates hyperemia in the brain). AVDO_2_ and CEO_2_, calculated using Fick’s equations, directly reflect the cerebral microcirculation and oxygen supply-demand (13,14). The results of the present study revealed that ulinastatin decreases the levels of JVBL and AVDO_2_ and increases the levels of SjvO_2_ and CEO_2_ in patients with sTBI. These results further demonstrate that ulinastatin improves cerebral ischemia-hypoxia and enhances the utilization of oxygen (15). A previous study reported that ulinastatin reduces the rate of mortality by decreasing gastrointestinal bleeding and protecting immunological and renal functions (16). The results of the present study demonstrated that the CRP level increased rapidly following sTBI and ulinastatin administration lowered the CRP level significantly. Less gastrointestinal bleeding and a lower mortality rate was observed in the observation group, indicating that ulinastatin is effective in the treatment of sTBI. The potential mechanism of ulinastatin with regard to the treatment of sTBI may involve the clearance of oxygen free radicals and inhibiting the release of lysosomal enzymes and inflammatory mediators (17).

In conclusion, ulinastatin administration effectively improves the prognosis of patients with sTBI by improving cerebral oxygen metabolism, lowering the CRP level and reducing gastrointestinal bleeding and the mortality rate.

## Figures and Tables

**Table I:** Comparison of cerebral oxygen metabolism parameters between the two groups.

Groups	Index	Day 1	Day 3	Day 5	Day 7
Observation	JVBL (mmol/l)	3.58±1.14	2.53±0.69^a^,^b^	1.46±0.53^a^,^b^	1.32±0.39^a^,^b^
	SjvO_2_ (%)	51.49±7.21	71.47±7.85^a^,^b^	68.21±5.48^a^,^b^	63.36±5.13^a^,^b^
	AVDO_2_ (ml/l)	69.18±14.32	39.56±11.39^a^,^b^	45.38±9.24^a^,^b^	48.26±12.43^a^,^b^
	CEO_2_ (%)	48.48±4.52	36.25±5.83^a^,^b^	39.13±5.23^a^,^b^	40.18±5.47^b^
Control	JVBL (mmol/l)	3.52±1.12	3.47±0.72	3.05±0.44	2.85±0.36
	SjvO_2_ (%)	50.83±7.05	61.52±6.59^a^	60.72±5.22^a^	54.27±5.06
	AVDO_2_ (ml/l)	67.55±13.40	49.41±12.18^a^	53.19±7.32^a^	55.45±13.30
	CEO_2_ (%)	48.39±4.43	25.19±5.07^a^	31.46±4.92^a^	32.43±4.15^a^

a P<0.05, vs. day 1 in the same group;

b P<0.05, vs. control group.

JVBL, jugular venous blood lactate; SjvO_2_, jugular venous bulb oxygen saturation; AVDO_2_, arteriovenous oxygen content difference; CEO_2_, cerebral extraction of oxygen.

**Table II:** Comparison of the CRP levels between the two groups (mg/l).

Groups	Day 1	Day 3	Day 5	Day 7
Observation (n=46)	32.15±10.28	51.46±11.39	35.27±15.18^a^	22.65±10.48^a^
Control (n=46)	31.49±10.07	47.22±12.36	56.19±13.24	47.36±15.73

a P<0.05, vs. control group.

CRP, C-reactive protein.
